# Global Perspectives on Regional Sun Protection Factor (SPF) Requirements: Scientific and Regulatory Insights

**DOI:** 10.7759/cureus.103757

**Published:** 2026-02-17

**Authors:** Maheshvari N Patel, Nayan Patel

**Affiliations:** 1 Clinical Research Operations, NovoBliss Research Private Limited, Ahmedabad, IND; 2 Pharmacology, Swaminarayan University, Ahmedabad, IND

**Keywords:** cosmetics, drug regulation, global regulatory guidelines, region-specific spf, sun protection factor (spf), sunscreens, ultraviolet rays

## Abstract

Ultraviolet (UV) radiation is a well-established environmental factor contributing to acute and chronic skin damage, including erythema, photoaging, pigmentary disorders, and skin cancer. Sunscreens represent a cornerstone of photoprotection strategies, with sun protection factor (SPF) serving as the primary measure of protection against UVB-induced erythema. However, UV exposure varies considerably across geographic regions due to differences in latitude, climate, altitude, and lifestyle, necessitating region-specific SPF recommendations. In parallel, sunscreen regulation and SPF claim substantiation differ across global jurisdictions, influencing product development, labeling, and consumer communication. This scientific communication reviews the biological basis of UV-induced skin damage, the rationale for region-wise SPF selection, and the regulatory frameworks governing sunscreen efficacy and claims across major global authorities, including India (Bureau of Indian Standards (BIS)), the European Union (EU; European Cosmetic, Toiletry and Perfumery Association (COLIPA)), the United States (Food and Drug Administration (FDA)), Australia (Therapeutic Goods Administration (TGA)), Canada (Health Canada), and Japan (Ministry of Health, Labour and Welfare (MHLW)/Japan Cosmetic Industry Association (JCIA)). While regulatory approaches differ in classification, UVA assessment methodologies, and labeling systems, there is a shared scientific consensus on the importance of validated SPF testing, broad-spectrum protection, and responsible communication. Harmonization through international testing standards has improved comparability of sunscreen efficacy, although regulatory diversity continues to pose challenges for global implementation. Integrating scientific evidence with existing region-specific regulatory requirements is essential for effective photoprotection, informed sunscreen use, and improved public health outcomes.

## Editorial

Solar ultraviolet (UV) radiation is a pervasive environmental exposure with major implications for skin health. While limited sunlight exposure supports vitamin D synthesis, excessive UV exposure is strongly associated with acute effects (erythema/sunburn) and chronic outcomes, including photoaging, pigmentary disorders, immunosuppression, and skin cancers. The World Health Organization (WHO) and the International Agency for Research on Cancer (IARC) recognize solar radiation and UV radiation as carcinogenic to humans, underscoring the public health importance of effective photoprotection strategies. Globally, skin cancer represents a significant and growing health burden, with more than 1.5 million new skin cancer cases reported worldwide in 2022. Among these, approximately 330,000-331,000 cases were diagnosed as melanoma, resulting in nearly 58,000-60,000 deaths annually, while non-melanoma skin cancers accounted for over 1.2 million new cases and approximately 69,000 deaths globally. These data highlight the substantial contribution of UV radiation exposure to skin cancer incidence and emphasize the importance of preventive strategies such as sunscreen use and photoprotection awareness programs [1,2]. 

The UV spectrum that reaches the Earth’s surface consists primarily of UVA (320-400 nm) and UVB (290-320 nm). UVB is more erythemogenic and is a principal driver of sunburn and direct DNA photodamage, whereas UVA penetrates deeper into the dermis, contributing substantially to oxidative stress, photoaging, and longer-term photodamage. Importantly, UVA exposure can be substantial during daily life and is less influenced by seasonality than UVB, reinforcing the need for broad-spectrum protection beyond UVB-focused metrics alone.

Topical sunscreens remain a cornerstone of photoprotection because they reduce UV radiation reaching viable epidermal and dermal targets. Sun protection factor (SPF) is the most widely used performance metric and is designed to quantify protection against UVB-induced erythema under standardized testing conditions. However, real-world sunscreen effectiveness is frequently lower than labeled values because many users apply less than the test dose and fail to reapply after sweating, swimming, or prolonged exposure. Consequently, selection of an appropriate SPF must be considered alongside broad-spectrum coverage, correct application practices, and behavioral sun-protection measures [3].

Evidence also supports sunscreen use as an effective preventive intervention. Long-term follow-up of a community-based randomized trial demonstrated reduced melanoma incidence associated with regular sunscreen use, strengthening the clinical rationale for routine photoprotection [4].

Despite global consensus on the value of sunscreen use, UV exposure is not uniform across geography. Latitude, altitude, climate, cloud cover, and lifestyle patterns produce meaningful regional variability in UV burden, which can influence the level of protection required for daily and occupational exposure. In addition to environmental factors, individual dermatological risk determinants also significantly influence susceptibility to photodamage. Populations with immunocompromised status, including organ transplant recipients and patients receiving immunosuppressive therapy, demonstrate an increased risk of UV-induced skin malignancies. Similarly, individuals with a prior history of skin diseases, including actinic keratosis, photosensitive dermatoses, or previous skin cancers, exhibit heightened vulnerability to UV radiation. Therefore, a region-specific approach to SPF recommendations, aligned with established regulatory frameworks and testing standards, is needed to guide clinicians, consumers, and product developers. This scientific communication aims to synthesize the biological rationale for region-differentiated SPF selection and to contextualize such recommendations within major regulatory and guideline systems. 

Ultraviolet radiation and geographic variability

Solar UV radiation is not a uniform environmental exposure; rather, it varies significantly across geographic regions due to differences in latitude, altitude, atmospheric conditions, and climatic factors. The two primary UV bands that reach the earth’s surface, UVA (320-400 nm) and UVB (290-320 nm), differ in their biological effects and are influenced differently by environmental paSkiprameters.

Types of UV radiation and biological impact 

UVA radiation represents the majority of UV energy reaching the earth’s surface and penetrates deeper into the dermis, causing indirect DNA damage via reactive oxygen species (ROS) and contributing to photoaging and immunosuppression. In contrast, UVB radiation has shorter wavelengths that are more energetic, directly inducing DNA photolesions such as cyclobutane pyrimidine dimers, which are strongly associated with erythema and carcinogenesis [5]. These mechanistic differences underscore the importance of broad-spectrum photoprotection that targets both UVB and UVA wavelengths.

Latitude and solar zenith angle

One of the principal determinants of ground-level UV irradiance is latitude. Regions closer to the equator experience a higher solar zenith angle, which reduces the distance sunlight must travel through the atmosphere, thereby increasing the intensity of UV radiation at the surface. Consequently, equatorial and tropical regions consistently record higher UV Index (UVI) values throughout the year compared to temperate regions.

For example, equatorial zones commonly have peak UVI values exceeding 10-11, which are categorized as “very high” by the WHO guidelines, compared to temperate regions where peak UVI typically ranges lower and is more seasonal.

Altitude and atmospheric conditions

Altitude also significantly influences UV exposure. For every 1000 meters increase in altitude, UV radiation increases by approximately 10% to 12%, due to thinner atmospheric layers filtering less radiation. High-altitude regions such as mountainous terrains therefore experience more intense UV exposure, even at higher latitudes. Moreover, atmospheric ozone concentration, cloud cover, and particulate matter can attenuate or enhance surface UV levels. For instance, reduced ozone leads to increased UVB, while dense cloud cover can decrease total UV irradiance [6, 7].

Seasonal and behavioral variability

Seasonal changes in Earth’s tilt also contribute to variability in UV exposure. During summer months, higher solar elevation angles result in increased UV irradiance, whereas winter months are associated with lower UVB levels, though UVA may remain relatively constant. Behavioral factors such as outdoor occupational activities, clothing practices, and cultural sun exposure habits contribute additional variability in individual UV dose independent of environmental UV metrics [8].

Implications for photoprotection strategies

The combined influence of latitude, altitude, seasonal shifts, and human behavior creates distinct UV exposure profiles across regions. These differences justify a regionally contextual photoprotection strategy that considers not only the quantitative UV burden but also the qualitative impacts of UVA versus UVB exposure. Consequently, sunscreen recommendations, including SPF and broad-spectrum designation, must be tailored according to geographic and lifestyle risk factors to effectively reduce acute and cumulative UV damage.

Scientific basis for regional SPF differentiation

The concept of regional differentiation in SPF recommendations is grounded in the quantitative and qualitative variability of UV radiation exposure and its biological effects on human skin. SPF is defined as the ratio of the minimal erythema dose (MED) on sunscreen-protected skin to the MED on unprotected skin and serves as a surrogate marker of protection against UVB-induced erythema. While SPF provides a standardized metric under controlled laboratory conditions, its clinical relevance is influenced by environmental exposure intensity, duration, and user behavior, all of which vary geographically [9, 10].

Relationship between UVB dose and erythema

UVB radiation is the primary determinant of acute erythema and is therefore the basis for SPF determination. Regions experiencing higher ambient UVB irradiance, such as tropical, equatorial, desert, and high-altitude areas, are associated with faster accumulation of erythemal dose, increasing the likelihood of sunburn during routine outdoor exposure. In such regions, higher SPF formulations provide a greater margin of protection against inadvertent under-application and prolonged exposure. Conversely, regions with lower UVB intensity may achieve adequate erythema protection with moderate SPF products when used appropriately [11].

Non-linear nature of SPF protection

Although SPF values are often perceived as linear indicators of protection, the relationship between SPF and UVB filtration is logarithmic. For example, SPF 15 filters approximately 93% of UVB radiation, SPF 30 filters about 97%, and SPF 50 filters approximately 98%. This non-linear increase implies diminishing returns at higher SPF values; however, in high-UV regions, even small incremental gains in UVB attenuation may be clinically meaningful, particularly in real-world conditions where sunscreen is under-applied.

Role of UVA exposure in regional risk

Unlike UVB, UVA radiation shows relatively less geographic and seasonal variability and remains present throughout the year. UVA penetrates deeper into the skin and contributes significantly to photoaging, pigmentary changes, and immunomodulation. In high-sunlight regions, cumulative UVA exposure is substantially greater due to longer daily exposure times, reinforcing the need for higher SPF products combined with robust UVA protection. Therefore, regional SPF differentiation must be considered alongside broad-spectrum requirements rather than SPF in isolation.

Skin phototype and population-level considerations

The population distribution of skin phototypes also influences regional SPF needs. Individuals with lighter skin phototypes (Fitzpatrick I-III) exhibit lower MED values and are more susceptible to UV-induced erythema and carcinogenesis, necessitating higher SPF protection, particularly in high-UV environments. In contrast, darker phototypes, while more resistant to erythema, remain vulnerable to UVA-mediated photoaging and pigmentary disorders, supporting the universal need for adequate SPF and UVA protection across all regions [12].

Behavioural and occupational modifiers

Geographic regions are often associated with distinct lifestyle and occupational exposure patterns. Outdoor occupations, coastal activities, and cultural clothing practices can significantly increase cumulative UV dose independent of ambient UVI. In such contexts, higher SPF recommendations serve as a practical risk-mitigation strategy to account for extended exposure duration and inconsistent reapplication practices.

Scientific rationale for region-specific SPF recommendations

Taken together, ambient UV intensity, spectral composition of radiation, population skin phototype distribution, and exposure behavior form the scientific basis for region-specific SPF differentiation. High-UV regions warrant routine use of high-SPF (SPF 50 or above) sunscreens, whereas moderate-UV regions may achieve effective protection with SPF 30-50, and lower-UV regions may rely on SPF 15-30 for daily exposure. Importantly, all regional recommendations must be integrated with broad-spectrum protection to address both acute and cumulative UV-induced skin damage.

Region-wise SPF requirements and recommendations 

Regional variation in UV radiation exposure necessitates differentiated SPF recommendations to ensure effective photoprotection. Geographic factors such as latitude, altitude, climate, and seasonal variability, combined with lifestyle and occupational exposure, determine the cumulative UV dose received by the skin. Consequently, a “one-size-fits-all” SPF recommendation may be inadequate to address region-specific photodamage risks.

High UV Exposure Regions

High UV exposure regions include tropical and equatorial zones, coastal areas, desert regions, and high-altitude terrains. These regions are characterized by persistently high UVI values, often exceeding 8-11 during peak hours, and prolonged daily sun exposure. In such environments, individuals accumulate erythemal doses rapidly, increasing the risk of acute sunburn, pigmentation, and long-term photodamage.

For these regions, SPF 50 or SPF 50+ sunscreens are recommended for routine daily use. Higher SPF values provide a greater safety margin against under-application and extended exposure, which are common in real-world settings. In addition to high SPF, broad-spectrum protection and water or sweat resistance are particularly important due to outdoor activities and climatic conditions such as heat and humidity.

Moderate UV Exposure Regions

Moderate UV exposure regions typically include subtropical zones, urban plains, and regions with seasonal variation in UV intensity. In these areas, UVI values fluctuate across the year, with higher exposure during summer months and reduced intensity during winter. Although the risk of acute erythema may be lower than in tropical regions, cumulative UV exposure remains clinically relevant [13].

For these regions, SPF 30-50 sunscreens are generally considered adequate for daily photoprotection. SPF 30 provides substantial UVB attenuation when applied correctly, while SPF 50 may be preferred during periods of prolonged outdoor exposure or peak summer conditions. Broad-spectrum coverage remains essential to mitigate UVA-induced photoaging and pigmentary changes.

Low to Moderate UV Exposure Regions 

Low to moderate UV exposure regions include temperate climates, higher latitude areas, and regions with frequent cloud cover. These areas experience lower annual UV indices, particularly during winter months. However, incidental daily exposure and reflective surfaces such as snow can still result in significant UV dose accumulation [14].

In these regions, SPF 15-30 sunscreens may be sufficient for routine daily activities, particularly for predominantly indoor lifestyles. Nonetheless, higher SPF products should be recommended during travel to high-UV regions, outdoor recreational activities, or extended sun exposure. Importantly, UVA protection remains necessary regardless of regional UVB intensity.

Special Considerations: Occupational and Lifestyle Exposure

Beyond geographic classification, occupational and behavioral factors significantly influence SPF requirements. Outdoor workers, athletes, and individuals engaged in recreational sun exposure may require higher SPF protection irrespective of the regional UV category. Similarly, children, individuals with photosensitive conditions, and those with lighter skin phototypes benefit from higher SPF formulations across all regions [15].

Summary of Region-Based SPF Recommendations

Collectively, scientific evidence supports a tiered approach to SPF selection based on regional UV exposure intensity: high-UV regions warrant SPF 50+, moderate-UV regions benefit from SPF 30-50, and lower-UV regions may rely on SPF 15-30 for daily use. These recommendations must be integrated with broad-spectrum protection, appropriate application, and reapplication practices to ensure real-world effectiveness.

SPF selection for routine exposure in India

India lies largely within the tropical and subtropical zones, resulting in moderate to high UV exposure across most regions for a significant part of the year. For routine daily activities involving incidental sun exposure, such as commuting, short outdoor durations, and predominantly indoor lifestyles, broad-spectrum sunscreen with an SPF of 30 is generally considered sufficient for effective photoprotection. SPF 30 blocks approximately 97% of UVB radiation when applied at the recommended dose and has been widely endorsed in dermatological literature as an appropriate balance between efficacy, cosmetic acceptability, and user compliance. Clinical guidance specific to Indian skin types (predominantly Fitzpatrick III-V) supports SPF 30 as an adequate daily photoprotective measure, particularly when combined with UVA protection, as it effectively reduces UVB-induced erythema and contributes to the prevention of photoaging and pigmentary disorders [12]. 

Regulatory guidelines governing sunscreen and SPF claims

Bureau of Indian Standards (BIS): India

In India, sunscreens are regulated as cosmetic products under the Drugs and Cosmetics Act, 1940, and the associated Cosmetics Rules, with quality and labeling requirements specified by the BIS (IS 17494:2021). BIS plays a central role in ensuring the safety, quality, and truthful representation of sunscreen products marketed in the country, including substantiation of SPF claims.

SPF Determination Requirements

BIS mandates that SPF claims on sunscreen products must be supported by scientifically validated testing methods. India follows internationally harmonized approaches for SPF evaluation, with in vivo SPF determination aligned with International Organization for Standardization (ISO) 24444: 2019, which is recognized globally as the reference standard for assessing protection against UVB-induced erythema. This method defines SPF as the ratio of the MED on protected skin to that on unprotected skin, with sunscreen application standardized at 2 mg/cm² under controlled conditions [16].

By adopting ISO-aligned methodologies, BIS ensures reproducibility, reliability, and international comparability of SPF values. This harmonization is particularly important for India, given its high ambient UV exposure and the widespread use of imported and locally manufactured sunscreen formulations. Products claiming specific SPF values must therefore demonstrate compliance with validated testing protocols and maintain consistency between labelled SPF and measured performance.

Labelling and Safety Considerations

BIS requires that sunscreen labelling be clear, non-misleading, and scientifically substantiated. SPF values must be explicitly declared on the product label, along with directions for use that enable consumers to achieve the intended level of protection. Labels are also expected to include appropriate warnings, usage instructions, and ingredient disclosures in accordance with IS 17494: 2021, which governs the labelling of cosmetic products in India [17].

From a safety perspective, BIS emphasizes compliance with permitted UV filter lists, concentration limits, and general cosmetic safety standards. Sunscreen formulations must not contain prohibited substances and should be evaluated for skin compatibility to minimize the risk of irritation or sensitization, particularly given the frequent and repeated use of such products in high-UV climates. While SPF primarily reflects UVB protection (IS 17492: 2021/ ISO 24442: 2019), BIS guidance increasingly aligns with international expectations for balanced photoprotection, encouraging inclusion of UVA protection (IS 17492: 2021/ ISO 24442: 2011) as part of responsible sunscreen formulation and labelling practices [17].

Overall, the BIS regulatory framework seeks to balance consumer safety, scientific validity of SPF claims, and harmonization with global standards, ensuring that sunscreens marketed in India provide reliable and effective photoprotection suited to local climatic conditions.

Permitted Sunscreen Ingredients under BIS (India)

Under the Indian cosmetic regulatory framework governed by the BIS and the Drugs and Cosmetics Act, 1940, sunscreens may contain both inorganic (physical) and organic (chemical) UV filters that are permitted within prescribed concentration limits. Commonly used inorganic UV filters include zinc oxide and titanium dioxide, which provide broad-spectrum protection through reflection and scattering of UV radiation. Organic UV filters permitted in India include UVB filters such as ethylhexyl methoxycinnamate, octocrylene, homosalate, and ethylhexyl triazone, UVA filters such as butyl methoxydibenzoylmethane, and broad-spectrum filters including bis-ethylhexyloxyphenol methoxyphenyl triazine and methylene bis-benzotriazolyl tetramethylbutylphenol. All sunscreen ingredients must comply with the latest provisions of IS 4707 (Part 1) and IS 4707 (Part 2), in accordance with the Drugs and Cosmetics Act, 1940, and Cosmetics Rules, 2020. The UV filters used in sunscreen formulations shall conform specifically to IS 4707 (Part 4). In addition, the safety evaluation of any novel ingredient used in sunscreen formulations shall be conducted in compliance with IS 4011, and all ingredients must be appropriately declared on product labels. SPF and broad-spectrum claims must be supported through scientifically validated testing methods [17].

European Cosmetic, Toiletry and Perfumery Association (COLIPA): European Union (EU) sunscreen guidelines

In the EU, sunscreens are regulated as cosmetic products under Regulation (EC) No. 1223/2009, with scientific guidance on efficacy claims and labelling historically developed by the COLIPA, now Cosmetics Europe. COLIPA guidelines have played a pivotal role in standardizing sunscreen efficacy evaluation, labelling, and consumer communication across EU member states and are widely regarded as a global benchmark for balanced photoprotection [18].

SPF Determination and UVA Protection Requirements

Under EU guidance, SPF determination follows standardized in vivo testing methods aligned with ISO 24444, ensuring reproducibility and harmonization across markets. Importantly, COLIPA introduced the principle that SPF alone is insufficient to describe overall photoprotection, emphasizing the need for mandatory UVA protection alongside UVB protection [19].

To ensure balanced photoprotection, EU guidelines require that the UVA protection factor be at least one-third of the labelled SPF value. UVA protection is assessed using validated in vitro methods such as ISO 24443, which evaluates UVA absorbance across the relevant wavelength range. This requirement directly addresses the role of UVA radiation in photoaging, immunosuppression, and long-term skin damage [20].

Protection Category Classification

COLIPA guidelines recommend classifying sunscreens into protection categories to improve consumer understanding and avoid misleading claims.

This categorical system discourages exaggerated SPF claims and reinforces the concept that no sunscreen provides complete protection. Claims such as “sunblock” or “total protection” are discouraged under EU guidance [19].

Labelling and Consumer Communication

EU regulations emphasize clear, honest, and non-misleading labelling. Sunscreen labels must indicate the SPF value, protection category, and usage instructions, including application quantity and reapplication frequency. COLIPA guidance explicitly advises against claims that could encourage prolonged sun exposure or give a false sense of security [19]. This consumer-centric regulatory philosophy aligns sunscreen communication with public health objectives rather than purely numerical SPF escalation.

Ingredient and Safety Considerations

The EU maintains a positive list of permitted UV filters (Annex VI of Regulation (EC) No. 1223/2009), with clearly defined maximum concentration limits and safety evaluations conducted by the Scientific Committee on Consumer Safety (SCCS). Only UV filters included in this annex may be used in sunscreen formulations marketed in the EU [19]. This rigorous pre-market safety assessment framework ensures both efficacy and consumer safety while enabling innovation within scientifically validated boundaries.

Regulatory Perspective

The COLIPA/EU framework represents one of the most comprehensive sunscreen regulatory systems globally, integrating SPF validation, mandatory UVA protection, controlled claims, and strict ingredient safety assessment. Its emphasis on balanced photoprotection and responsible communication has strongly influenced sunscreen regulations worldwide and serves as a reference model for harmonization across international markets.

United States Food and Drug Administration (FDA) sunscreen guidelines

In the United States, sunscreens are regulated as over-the-counter (OTC) drug products by the FDA under the Federal Food, Drug, and Cosmetic Act. Unlike many regions where sunscreens are classified as cosmetics, the FDA’s drug-based regulatory approach places strong emphasis on safety, efficacy, standardized testing, and consumer risk communication.

SPF Determination and UVB Protection

The FDA defines SPF as a measure of protection against UVB-induced erythema and requires SPF determination through standardized in vivo testing conducted under controlled conditions. The FDA’s SPF testing methodology is conceptually aligned with international standards, using MED ratios following application at 2 mg/cm². SPF values are permitted to be labelled only when substantiated by FDA-recognized testing protocols, and the FDA emphasizes that SPF values reflect protection against UVB radiation alone [21].

Importantly, the FDA restricts exaggerated interpretations of SPF by regulating how SPF values are presented and understood, reinforcing that higher SPF does not equate to complete protection from solar radiation [21].

Standards Governing Protection Requirements

The FDA mandates that sunscreens may only be labelled as “broad spectrum” if they demonstrate significant protection against both UVA and UVB radiation. Broad-spectrum efficacy is assessed using a wavelength-based in vitro test that evaluates UVA absorbance across the 320-400 nm range. Products that fail to meet broad-spectrum criteria must carry a warning stating that they protect only against sunburn and do not reduce the risk of skin cancer or early skin aging [21].

This regulatory requirement reflects the FDA’s recognition of UVA radiation as a contributor to photoaging and skin cancer, while maintaining a clear distinction between UVB-related SPF claims and UVA protection.

Ingredient Regulation and Safety Assessment

The FDA regulates sunscreen active ingredients through a monograph system, classifying UV filters based on whether they are generally recognized as safe and effective (GRASE). Ingredients that lack sufficient safety or efficacy data are subject to additional evaluation or restrictions. This framework underscores the FDA’s conservative, evidence-driven approach to sunscreen ingredient approval, prioritizing long-term consumer safety [21].

Regulatory Perspective

The FDA sunscreen framework is distinguished by its drug-level regulatory rigor, focus on UVB-based SPF accuracy, mandatory broad-spectrum substantiation, and strong consumer risk communication. While more restrictive than cosmetic-based systems, the FDA approach provides a high level of assurance regarding safety and claim validity, and continues to influence global discussions on sunscreen efficacy evaluation and labeling standards.

Australian Therapeutic Goods Administration (TGA) sunscreen guidelines

In Australia, sunscreens are regulated by the TGA under the TGA 1989, reflecting the country’s high ambient UV radiation levels and elevated skin cancer risk. Unlike regions where sunscreens are regulated solely as cosmetics, Australia adopts a risk-based therapeutic regulatory approach, recognizing sunscreens as public health interventions rather than purely cosmetic products.

Regulatory Classification of Sunscreens

The TGA classifies sunscreens into primary and secondary sunscreen products. Primary sunscreens, whose principal function is UV protection, are regulated as therapeutic goods and must be included in the Australian Register of Therapeutic Goods (ARTG) before marketing. Secondary sunscreens, in which UV protection is a secondary function (e.g., moisturizers or cosmetics with SPF), are also subject to regulatory oversight, though with proportionate requirements. This classification ensures that products making photoprotective claims meet appropriate safety and efficacy standards [22].

SPF Determination and Performance Requirements

Australia mandates that SPF claims be supported by validated in vivo testing methods, aligned with internationally recognized standards such as AS/NZS 2604 (Sunscreen Products-Evaluation and Classification). SPF values reflect protection against UVB-induced erythema, and testing must be conducted under standardized conditions using a defined application dose. Given Australia’s extreme UV environment, the TGA places particular emphasis on accurate SPF substantiation and reproducibility of results [22].

Broad-Spectrum Protection Requirements

The TGA requires that sunscreens labeled as “broad spectrum” provide effective protection against both UVA and UVB radiation. Broad-spectrum designation is permitted only when the sunscreen demonstrates a minimum UVA protection level, assessed using validated test methods consistent with AS/NZS standards. This requirement addresses the significant contribution of UVA radiation to photoaging, immunosuppression, and skin cancer risk in high-UV environments such as Australia [23].

Ingredient Safety and Compliance

Only approved UV filters and excipients may be used in Australian sunscreen formulations, and all active ingredients must meet stringent safety and quality requirements. The TGA requires robust safety data to support ingredient use, reflecting Australia’s precautionary regulatory philosophy. This ensures that sunscreens used frequently and over large body surface areas maintain a favorable risk-benefit profile.

Regulatory Perspective

The Australian TGA sunscreen framework represents one of the most rigorous global regulatory models, integrating therapeutic-level efficacy testing, mandatory broad-spectrum protection, and strong consumer risk communication. Given Australia’s high UV burden and skin cancer prevalence, this regulatory approach underscores the role of sunscreens as essential preventive healthcare products rather than optional cosmetic aids.

Health Canada sunscreen guidelines

In Canada, sunscreen products are regulated by Health Canada as drug products under the Food and Drugs Act and Regulations. These regulations ensure that sunscreens authorized for sale deliver safe and effective photoprotection against UV radiation and that their labelling reflects scientifically validated claims.

Regulatory Framework and Product Authorization

Health Canada regulates sunscreens through a drug authorization process that evaluates safety, efficacy, and quality before products can be sold. Depending on the formulation and medicinal ingredient(s) used, sunscreens may be classified as non-prescription drugs (NPDs) or natural health products (NHPs), each requiring an appropriate authorization number, such as a Drug Identification Number (DIN) or a Natural Product Number (NPN). This regulatory classification influences requirements for testing, labelling, and post-market surveillance [24].

SPF and Broad-Spectrum Protection Requirements

Health Canada recommends that consumers choose sunscreens with broad-spectrum protection, which indicates protection against both UVA and UVB radiation. “Broad-spectrum” labeling on Canadian sunscreen products signifies that the formulation provides substantive attenuation of both UVB (primarily responsible for erythema/sunburn) and UVA (associated with photoaging and long-term photodamage).

The SPF must be clearly stated on the product label, and testing must support the claimed SPF value. Health Canada’s guidance indicates that sunscreens with a broad-spectrum SPF value of 15 or higher are suitable for general use to help prevent sunburn and contribute to overall sun safety when combined with other protective measures such as shade and protective clothing.

Health Canada advises that sunscreen should be applied 15 minutes before sun exposure and reapplied at least every two hours, particularly after swimming, sweating, or towel drying, to maintain effective protection [25]. 

Safety and Post-market Surveillance

Health Canada conducts safety reviews of sunscreen products to monitor adverse reactions and ensure ongoing compliance. A 2018 review reaffirmed that the use of broad-spectrum sunscreens with SPF values of 30 or higher is recommended to prevent sunburn and reduce the risk of skin cancer, and that reported skin reactions are rare relative to the widespread benefits of sunscreen use [26].

Regulatory Perspective

Health Canada’s sunscreen regulations reflect a public health approach to UV protection, emphasizing both broad-spectrum efficacy and consumer education through labelling. By requiring clear SPF values, validated broad-spectrum claims, and user guidance on application and reapplication, Health Canada seeks to support effective photoprotection while minimizing the risk of misleading claims or misuse.

Japanese sunscreen guidelines

In Japan, sunscreen products are primarily regulated as cosmetics under the Standards for Cosmetics established by the Ministry of Health, Labour and Welfare (MHLW). While not mandated by law in the same way as therapeutic drug regulations in some countries, industry standards developed by the Japan Cosmetic Industry Association (JCIA) serve as widely accepted reference frameworks for evaluating sunscreen efficacy, labelling, and safety in the Japanese market. These standards are recognized by regulators and manufacturers alike to ensure consistency in sun protection claims [27].

SPF Testing and UVB Protection

Japanese sunscreen evaluation follows internationally harmonized methods for determining SPF. The JCIA standards adopt the in vivo SPF test method that is based on ISO 24444, aligning Japan with global practices for measuring protection against UVB-induced erythema. SPF values on product labels reflect the degree of UVB attenuation, and products may be labeled with specific SPF values from SPF 2 up to SPF 50, with higher protection indicated as SPF 50+ when appropriate [28].

UVA Protection and Protection Grade of UVA (PA) Rating System

Japan employs a PA rating system to communicate the extent of UVA protection. This system, developed domestically and now widely used in Asian markets, categorizes UVA effectiveness based on the persistent pigment darkening (PPD) method, with grading expressed in Table 1.

**Table 1:** PA Rating System for UVA Protection Source: [29] PA: Protection Grade of UVA; UVA: ultraviolet A

PA Grade	Level of UVA Protection
PA+	Some UVA protection
PA++	Moderate UVA protection
PA+++	High UVA protection
PA++++	Very high UVA protection

The PA system provides a straightforward visual indicator of UVA defense, complementing SPF values on product labels to convey broad-spectrum performance [29].

Standards and Voluntary Compliance

Domestic sunscreen evaluation in Japan is guided by JCIA voluntary standards, which are widely adopted by manufacturers and recognized by the regulatory authority. These standards outline methodologies for determining SPF, PA values, and water resistance for UV protection, and they are periodically updated to remain aligned with international norms such as ISO 24442 for UVA efficacy. Although voluntary, compliance with JCIA standards is considered best practice and supports consumer trust in product efficacy.

Labelling and Consumer Information

Sunscreen products marketed in Japan must comply with cosmetic labelling requirements under the Standards for Cosmetics. Labels typically include: SPF value for UVB protection, PA rating for UVA protection, directions for use, and reapplication

Claims must be truthful and substantiated; exaggerated or misleading protection claims (e.g., implying “complete protection”) are discouraged in alignment with both JCIA guidance and general cosmetic regulation.

Regulatory Perspective

Japan’s sunscreen regulatory approach balances international methodological harmonization with regionally tailored communication systems like the PA rating. Although regulations are not as prescriptive as therapeutic frameworks elsewhere, the combination of national cosmetic standards and industry consensus guidelines ensures that SPF and UVA protection claims are scientifically supported and consistently communicated to consumers (Table 2).

**Table 2:** Regulatory Classifications for SPF BIS: Bureau of Indian Standards; ISO: International Organization for Standardization; SPF: sun protection factor; UV: ultraviolet; EU: European Union; COLIPA: European Cosmetic, Toiletry and Perfumery Association; OTC: over the counter; FDA: Food and Drug Administration; PPD: persistent pigment darkening; TGA: Therapeutic Goods Administration; MHLW: Ministry of Health, Labour and Welfare; JCIA: Japan Chemical Industry Association; PA: Protection Grade of UVA

Region / Authority	Regulatory Classification	SPF Determination	UVA Protection Requirement	Labelling System	Key Regulatory Focus	Max SPF Requirement / Recommendation	Strength / Limitation	References
India (BIS)	Cosmetics	ISO-aligned in vivo SPF testing	Encouraged; aligned with international standards	SPF value, ingredient disclosure, usage instructions	Safety, truthful labelling, global harmonization	No defined maximum SPF cap; higher SPF permitted with substantiation	Strength: Alignment with global ISO testing. Limitation: Less prescriptive UVA performance threshold compared with EU	[16,17]
EU (COLIPA)	Cosmetics	ISO 24444 (in vivo)	Mandatory; UVA ≥ 1/3 of SPF (ISO 24443)	SPF + Protection category (Low–Very High)	Balanced UVA/UVB protection, consumer clarity	SPF values above 50 labelled as “SPF 50+	Strength: Scientifically balanced UVA-UVB requirement. Limitation: Categorical SPF labeling may reduce numeric differentiation	[18-20]
United States (FDA)	OTC Drug	FDA-defined in vivo SPF testing	Mandatory for “Broad Spectrum” (critical wavelength test)	SPF + Broad Spectrum + warnings	Consumer risk communication, strict claims control	No strict SPF cap; values may exceed SPF 50+ if substantiated	Strength: Strong claim and safety regulation. Limitation: Less quantitative UVA protection ratio requirement	[21]
Australia (TGA)	Therapeutic Goods	AS/NZS 2604–compliant SPF testing	Required for broad-spectrum claims	SPF, usage instructions, water resistance	Public health protection in high-UV environments	SPF 50+is typically considered the highest labelled category	Strength: Highly protective regulatory framework tailored to high UV exposure. Limitation: More complex regulatory pathway	[22]
Canada (Health Canada)	Drug / Natural Health Product	Validated SPF testing	Required for broad-spectrum labelling	SPF, broad spectrum, reapplication guidance	Public health–focused sun safety	SPF 50+ is typically the maximum marketed category	Strength: Strong consumer safety and risk communication. Limitation: Dual classification pathways may increase regulatory complexity	[24,25]
Japan (MHLW / JCIA)	Cosmetics	ISO-aligned in vivo SPF testing	PA rating system (PPD-based UVA grading)	SPF + PA (+ to ++++)	Simplified consumer communication, UVA emphasis	SPF is commonly labelled up to SPF 50+ with detailed UVA grading through the PA system	Strength: Clear UVA grading system (PA scale). Limitation: The PA system is less intuitive outside Asian markets	[28]

Comparative Analysis of Global SPF Regulations

Although sunscreen regulations across different regions are guided by a shared scientific understanding of UV radiation-induced skin damage, their regulatory implementation varies considerably. International authorities consistently recognize SPF as a measure of protection against UVB-induced erythema and acknowledge the importance of UVA protection for preventing photoaging and long-term photodamage. However, differences exist in regulatory classification, UVA assessment methodologies, labelling practices, and claim substantiation requirements. 

Conclusion 

SPF remains a validated and widely accepted measure of protection against UVB-induced erythema; however, effective photoprotection extends beyond SPF alone and requires consideration of UVA protection, exposure patterns, wavelength, PA grading, and geographic context. In India, where much of the population resides in tropical and subtropical regions with moderate to high ultraviolet exposure for most of the year, as per the UVI, routine photoprotection is an essential preventive measure. For daily, incidental sun exposure typical of indoor lifestyles and routine outdoor activities, broad-spectrum sunscreens with SPF 30 are generally sufficient, provided they are applied appropriately and used consistently. Higher SPF values may be reserved for prolonged outdoor exposure, occupational sun exposure, or high-UV environments, rather than routine daily use. When compared with global regulatory and clinical practices, the Indian approach to routine SPF use aligns closely with international recommendations as per the Indian Association of Dermatologists, Venereologists, and Leprologists. Regulatory authorities worldwide, including those in the European Union, the United States, Australia, Canada, and Japan, recognize SPF 30 as an effective baseline for daily photoprotection, emphasizing correct application, reapplication, and balanced UVA protection over escalation of SPF numbers alone. 

From a public health and regulatory perspective, defining an appropriate SPF range for India, rather than advocating universally high SPF values, may support rational sunscreen use and improved adherence. An evidence-based range of SPF 30-50 for Indian climatic conditions appears to accommodate routine daily exposure as well as higher-risk outdoor scenarios while remaining consistent with global scientific principles and regulatory observations. Aligning regional SPF considerations with international standards supports effective photoprotection and responsible consumer communication and highlights the role of sunscreens as an important preventive measure against UV-induced skin damage. 
